# The Emerging Roles of Circular RNAs in the Chemoresistance of Gastrointestinal Cancer

**DOI:** 10.3389/fcell.2022.821609

**Published:** 2022-01-21

**Authors:** Man Wang, Fei Yu, Yuan Zhang, Lei Zhang, Wenguang Chang, Kun Wang

**Affiliations:** Institute for Translational Medicine, The Affiliated Hospital of Qingdao University, College of Medicine, Qingdao University, Qingdao, China

**Keywords:** gastrointestinal cancer, chemoresistance, circular RNAs, molecular sponges, microRNAs, therapeutic targets

## Abstract

Gastrointestinal (GI) cancer represents a major global health problem due to its aggressive characteristics and poor prognosis. Despite the progress achieved in the development of treatment regimens, the clinical outcomes and therapeutic responses of patients with GI cancer remain unsatisfactory. Chemoresistance arising throughout the clinical intervention is undoubtedly a critical barrier for the successful treatment of GI cancer. However, the precise mechanisms associated with chemoresistance in GI cancer remain unclear. In the past decade, accumulating evidence has indicated that circular RNAs (circRNAs) play a key role in regulating cancer progression and chemoresistance. Notably, circRNAs function as molecular sponges that sequester microRNAs (miRNAs) and/or proteins, and thus indirectly control the expression of specific genes, which eventually promote or suppress drug resistance in GI cancer. Therefore, circRNAs may represent potential therapeutic targets for overcoming drug resistance in patients with GI cancer. This review comprehensively summarizes the regulatory roles of circRNAs in the development of chemoresistance in different GI cancers, including colorectal cancer, gastric cancer and esophageal cancer, as well as deciphers the underlying mechanisms and key molecules involved. Increasing knowledge of the important functions of circRNAs underlying drug resistance will provide new opportunities for developing efficacious therapeutic strategies against GI cancer.

## 1 Introduction

Gastrointestinal (GI) cancers are among the most important causes of cancer-related death worldwide and mainly include colorectal cancer (CRC), gastric cancer (GC) and esophageal cancer (EC) (Wang D.-K. et al., 2021). These GI cancers pose a huge threat to human health worldwide (Fang et al., 2021). Conventional treatments, including chemotherapy, radiotherapy and surgery, have been the mainstays of cancer therapy (Buckley et al., 2020). However, these therapeutic approaches have limitations and lead to unsatisfactory clinical outcomes, as evidenced by the high mortality rate of patients with GI cancers (D'Eliseo and Velotti, 2016; Hsu et al., 2020). Chemoresistance is a significant factor accounting for treatment failure in patients with GI cancer (Wang Y. et al., 2020). Therefore, a comprehensive investigation of the detailed mechanisms underlying cancer chemoresistance is essential to improve the efficacy of chemotherapy against GI cancer.

To date, a number of studies have suggested a linkage between noncoding RNAs (ncRNAs) and chemoresistance in GI cancer. Circular RNAs (circRNAs) are a class of endogenous ncRNA molecules with a covalently closed loop configuration (Patop et al., 2019). They are produced from exons and/or introns via the back-splicing pattern. CircRNAs were initially regarded as mis-splicing products without any genuine function and have not been adequately explored until recently (Sanger et al., 1976). The development of high-throughput sequencing technology and associated bioinformatics enables a thorough investigation of circRNAs. CircRNAs have been shown to play a crucial role in the chemoresistance of GI cancer. For instance, the circRNA circDDX17 sensitized CRC cells to 5-fluorouracil (5-FU) and promoted apoptosis via the miR-31-5p/kidney ankyrin repeat-containing protein 1 (KANK1) axis (Ren et al., 2020). CircHIPK3 acted as a molecular sponge for miR-637 to stimulate the downstream signal transducer and activator of transcription 3 (STAT3)/B-cell lymphoma-2 (Bcl-2)/Beclin 1 signaling cascade in CRC cells (Zhang et al., 2019). This event resulted in the suppression of autophagic cell death and enhanced oxaliplatin (OXA) resistance in CRC cells. However, current research aiming to understand the molecular mechanisms by which circRNAs regulate GI cancer chemoresistance is still in its initial stage. Further studies are warranted to enrich our knowledge of ncRNA-mediated mechanisms related to drug resistance in different GI cancers. In this review, we summarize the most recent evidence for the roles of circRNAs in regulating the drug resistance of GI cancer and discuss possible mechanisms of action. Future directions are suggested to better apprehend their exact roles and their usefulness as therapeutic targets.

## 2 Circular RNAs

Circular RNAs (circRNAs) are a class of single-stranded closed-loop RNA molecules that lack free 5′ and 3′ ends. Although circRNAs were first identified in viruses in 1976, they were originally considered splicing intermediates or byproducts of aberrant mRNA splicing events without specific functions and thus did not attract considerable scientific attention for many years (Sanger et al., 1976). With the advent of high-throughput sequencing techniques, a multitude of endogenous circRNAs have been discovered in various species. CircRNAs exhibit cell type-, tissue- or developmental stage-specific expression patterns, suggesting their possible biological significance. Accumulating evidence has verified that circRNAs are vital players in the initiation and progression of multiple human diseases, especially cancer (Verduci et al., 2021).

### 2.1 Biogenesis of Circular RNAs

**FIGURE 1:**
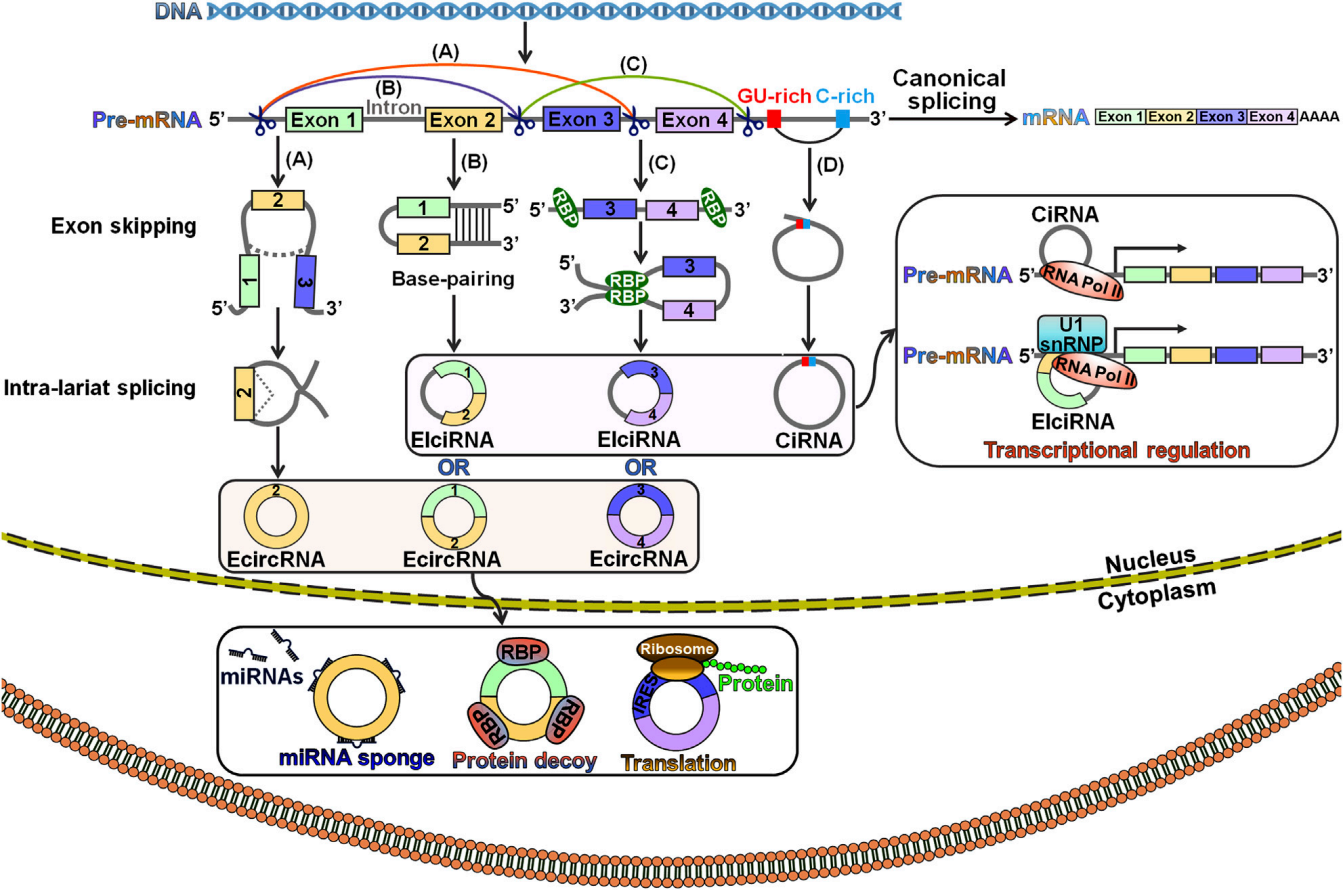
Schematic illustration of the biogenesis and functionality of circRNAs. **(A)** Lariat-driven circularization. A lariat containing exons is generated following the exon-skipping event. The lariat is then internally spliced, thus giving rise to an ecircRNA. **(B)** Intron pairing-driven circularization. Pre-mRNA flanking introns harbor complementary sequences or inverted repeats. The base-pairing of both sides of the introns leads to the creation of a circular structure. In this case, circRNAs can be grouped into ecircRNAs and EIciRNAs depending on whether the intronic sequences reside in the loop. **(C)** RBP-mediated circulation. RBPs can specifically interact with flanking introns that bring the back-spliced exons close together and promote the circulation. As a result, ecircRNAs or EIciRNAs are generated. **(D)** Biogenesis of ciRNA. CiRNAs are formed through a lariat-derived mechanism, which predominantly rely on conserved motifs encompassing a 7 nt GU-rich element near the 5′ splice site and an 11 nt C-rich element near the branch point within the introns. EIciRNAs and ciRNAs mainly locate in the nucleus, while the majority of ecircRNAs are exported to the cytoplasm. EIciRNAs and ciRNAs function to modulate the transcription of parental genes, while ecircRNAs can serve as miRNA sponges, protein decoys or templates for protein translation. Pre-mRNA, precursor mRNA; ecircRNA, exonic circRNA; miRNAs, microRNAs; EIciRNA, exon-intron circRNA; RBP, RNA-binding protein; IRES, internal ribosome entry site; ciRNA, circular intronic RNA; RNA Pol II, RNA polymerase II; U1 snRNP, U1 small nuclear ribonucleoprotein.

The exact mechanisms of circRNA biogenesis remain largely unknown. CircRNAs are generated from precursor mRNAs (pre-mRNAs) via a back-splicing process, which attaches a downstream splice donor (5′ splice site) to an upstream splice acceptor (3′ splice site) (Wilusz, 2018). The pre-mRNA is spliced into linear RNA by deleting introns. In contrast, back-splicing occurs in an inverse orientation to produce an RNA molecule with a single or multiple exons (Zhang et al., 2016). CircRNAs are derived from exons, introns, untranslated regions (UTRs), antisense transcripts and intergenic regions (Memczak et al., 2013; Lu et al., 2015). According to previous studies, canonical splicing signals and spliceosome machinery were required for circRNA biogenesis (Ashwal-Fluss et al., 2014; Liang et al., 2017). However, canonical splicing is a primary choice for gene splicing under most circumstances. Depletion of core spliceosome components resulted in the shift from linear mRNA production toward preferred output of circRNAs (Liang et al., 2017). Based on their distribution and biogenesis, circRNAs are categorized into three main subclasses: exonic circRNAs (ecircRNAs), exon-intron circRNAs (EIciRNAs) and circular intronic RNAs (ciRNAs) (Ashwal-Fluss et al., 2014; Chen et al., 2015; Kelly et al., 2015). EIciRNAs and ciRNAs mainly reside in the nucleus, while the majority of ecircRNAs are exported to the cytoplasm (Zhang et al., 2013; Li et al., 2015; Huang et al., 2018). Three classical models for circRNA biogenesis have been proposed, including lariat-driven circularization, intron pairing-driven circularization and RNA-binding protein (RBP)-mediated circularization (Liang and Wilusz, 2014; Barrett et al., 2015; Conn et al., 2015) (Figure 1).

Lariat-driven circularization is also known as the exon-skipping mechanism (Wu et al., 2019). In this model, exon skipping occurs during canonical splicing (Figure 1A). Specifically, the folding of the pre-mRNA during transcription brings the 5′ donor site of the upstream intron and the 3′ receptor site of the downstream intron into closer proximity, leading to the formation of a linear RNA with skipped exons and an exon-containing lariat precursor. This lariat precursor then undergoes internal back-splicing to produce a circRNA.

Intron pairing-driven circulation, also known as the direct back-splicing mechanism, is mediated by cis-acting regulatory elements that contain reverse complementary motifs specifically located in the flanking introns, leading to direct base pairings of flanking introns (Liang and Wilusz, 2014) (Figure 1B). Flanking complementary sequences (e.g., Alu sequences) play a critical role in exon circularization (Dubin et al., 1995). In the RBP-mediated circularization process, RBPs recognize and dock on particular sequence motifs of bordering introns (Figure 1C). This docking brings the flanking introns of the back-spliced exons close together, hence facilitating exon circularization. Muscleblind (MBL) and quaking (QKI) proteins promoted the formation of circMbl and circQKI by interacting with specific intronic motifs, respectively (Ashwal-Fluss et al., 2014; Conn et al., 2015). In all the models, the intronic segments are completely removed or retained to form ecircRNAs or EIciRNAs, respectively (Bahn et al., 2015).

During canonical splicing, introns are normally excised in lariat forms, which are quickly debranched and degraded by exonucleolytic enzymes (Cao et al., 2021). However, the excised intronic lariats occasionally escape debranching and degeneration processes, and thus form stable ciRNAs with a 2′,5′-phosphodiester bond between the splicing donor and the branchpoint (Figure 1D). A consensus motif containing both a 7 nt GU-rich element near the 5′ splice site and an 11 nt C-rich element adjacent to the branchpoint site is essential for ciRNA biogenesis (Zhang et al., 2013).

The biogenesis of circRNAs is a highly orchestrated process in which many cis/trans-acting elements are involved. The explicit mechanisms by which these regulators control circRNA abundance are worthy of further study. More cis/trans-regulators implicated in circRNA biogenesis remain to be characterized. Genetic or epigenetic factors have been proposed to regulate circRNA formation. Genomic abnormalities, such as short nucleotide variants and chromosome translocation, may cause sequence alterations in DNA segments flanking the encircled region, hence influencing the generation of circRNAs (Sudmant et al., 2015). Epigenetic modifications within genes and histones affect alternative splicing and may indirectly influence circRNA formation (Bentley, 2014). The epigenetic mechanisms contributing to circRNA formation warrant more detailed exploration.

### 2.2 Biological Functions of Circular RNAs

CircRNAs have emerged as crucial participants in various physiological and pathological processes (Ely et al., 2021). Currently, circRNAs have been the spotlight of cancer research, as numerous studies have revealed pivotal biological roles for various circRNAs in cancer. CircRNAs affect various hallmarks of cancer by functioning as miRNA sponges, interacting with RBPs, governing transcription and splicing and encoding functional peptides or proteins (Li et al., 2020) (Figure 1). Among these functions, miRNA sponging is the best-characterized regulatory mechanism for circRNAs. For instance, the circRNA ciRS-7 served as a molecular sponge for tumor-suppressive miR-7 and regulated the progression of various cancers, including esophageal squamous cell carcinoma (ESCC), GC and non-small-cell lung cancer (NSCLC) (Li R.-c. et al., 2018; Pan et al., 2018; Su et al., 2018). CircRNAs are capable of interacting with RBPs. The circRNA *cIARS* abrogated alkylation repair homologue 5 (ALKBH5)-mediated autophagy inhibition in hepatocellular carcinoma (HCC) by physically interacting with ALKBH5 (Liu Z. et al., 2020). CircZKSCAN1 inhibited the malignant characteristics of HCC cells by binding the RBP fragile X mental retardation protein (FMRP) to block the Wnt/β-catenin signaling pathway (Zhu et al., 2019). As cellular proteins affect multiple hallmarks of cancer, circRNAs indirectly regulate cancer development by altering protein structure and function through their functions as protein decoys, recruiters or scaffolds. Intriguingly, the roles of circRNAs in cancer may be partially attributed to their regulation of transcription and splicing. For example, oncogenic circERBB2 modulated ribosomal DNA transcription, a key step in ribosome biogenesis and cell proliferation, by altering the nucleolar localization of proliferation-associated protein 2G4 (PA2G4) (Huang et al., 2019a). As a result, circERBB2 exerted a positive effect on gallbladder cancer cell proliferation. CircNOL10 retarded the proliferation and promoted the apoptosis of lung cancer cells by enhancing the transcriptional regulatory effect of sex comb on midleg-like 1 (SCML1) on the humanin (HN) polypeptide family (Nan et al., 2019). CircURI1 was shown to inhibit GC metastasis (Wang et al., 2021d). In terms of mechanism, circURI1 dominated the alternative splicing of cell migration-related genes by directly binding heterogeneous nuclear ribonucleoprotein M (hnRNPM). Additionally, some circRNAs contain open reading frames (ORFs) that can be translated into peptides or proteins with key biological importance. CircRNA-encoded proteins serve as oncoproteins or tumor suppressors in cancer. CircPPP1R12A encoded the functional protein circPPP1R12A-73aa, which facilitated the growth and metastasis of colon cancer via the Hippo/yes-associated protein (YAP) signal transduction cascade (Zheng et al., 2019). Experimental evidence revealed that circFNDC3B-218aa encoded by circFNDC3B prevented colon cancer progression by limiting Snail expression (Pan et al., 2020). The biological functions of circRNA-encoded proteins have not yet been completely defined. Studies aiming to clarify whether circRNA-produced proteins and their full-length protein counterparts encoded by the linear transcript have the same capabilities are essential. The modulatory mechanisms underlying circRNA translation deserve continued study.

Altogether, circRNAs play multifaceted roles in cancer development and progression through diverse mechanisms. Presumably, particular circRNAs may simultaneously perform multiple functions and modulate the same cancer-related pathway through different modes of action. The identification of the potential concerted mechanisms of circRNAs might help provide a comprehensive landscape of oncogenic signaling cascades. On the other hand, one circRNA may be implicated in various cancer-associated processes by employing the same mechanism of action. Thus, significant work is needed to reveal the complex relationship between circRNAs and cancer pathogenesis. In addition, many important questions in relation to circRNA function remain unanswered. CircRNAs have been reported to interact with miRNAs and RBPs. Researchers have not yet determined whether circRNAs bind other types of ncRNAs or molecules. The effect of circRNAs on the translocation of cellular components (e.g., RNAs and proteins) is also unclear. Increased efforts are needed to systematically characterize the versatile functionalities of circRNAs. In-depth investigations of circRNAs would foster the development of effective approaches for cancer diagnosis and treatment.

### 2.3 Circular RNA Expression Profiles in Gastrointestinal Cancer

With the help of circRNA microarray analysis and high-throughput sequencing technology, various deregulated circRNAs have been discovered in GI cancer, suggesting their important functions in the occurrence and development of GI cancer. For instance, 21,458 circRNAs were discovered in four paired CRC tissues and adjacent normal mucosa tissues using high-throughput RNA sequencing (RNA-seq) (Li X.-N. et al., 2018). Further analysis indicated that 448 circRNAs were differentially expressed in CRC tissues compared with normal mucosa tissues, including 394 upregulated and 54 downregulated circRNAs. These differentially expressed circRNAs were involved in the regulation of cell communication, autophagosome and GTPase binding. They might also be correlated with CRC-relevant signaling pathways, such as deleted in colorectal carcinoma (DCC)-mediated attractive signaling and the Netrin-1 signaling pathway. Accordingly, circRNAs played an important role in CRC carcinogenesis. The circRNA-seq data from 40 CRC samples revealed 113 (92 upregulated and 21 downregulated) dysregulated circRNAs in CRC patients with liver metastasis compared with CRC patients (Xu et al., 2019). In particular, two upregulated circRNAs, circRNA_0001178 and circRNA_0000826, presented potential diagnostic value in CRC patients with liver metastasis. By performing RNA-seq in combination with bioinformatics analysis, Sun Y. et al. (2020) identified 1,055 circRNAs that were abnormally expressed in three pairs of ESCC and adjacent normal tissues, 418 of which were upregulated and 637 were downregulated. Circ_0000075, circ_0000513, circ_0000530, circ_0001005, circ_0001121, circ_0001904 and circ_0002255 were predicted to be key circRNAs involved in ESCC pathogenesis. These circRNAs might be involved in ESCC progression through regulation of adherens junction and angiogenesis. A systematic meta-analysis based on circRNA microarrays revealed 64 differentially expressed circRNAs between GC tissues and adjacent normal tissues (Ding et al., 2020). Among these, hsa_circ_0005927 and hsa_circ_0067934 were identified as potential biomarkers for GC screening. The expression profile of circRNAs in liver cancer was previously assessed (Wang M. et al., 2020). Based on circRNA-seq data and bioinformatics analysis, 13,124 unique circRNAs were identified in three paired liver cancer tissues and adjacent normal tissues, 2,996 of which showed different expression patterns. These abnormally expressed circRNAs might exert regulatory effects on tumor growth and immunity in liver cancer. In another study, by performing circRNA-seq of 30 primary HCC tissues, Hu et al. (2020) detected 72,277 circRNAs that were expressed in at least one sample. The authors further screened 144 upregulated and 76 downregulated circRNAs in metastatic HCC compared with non-metastatic HCC, indicating their potential association with HCC metastasis. The dysregulated circRNAs in pancreatic cancer were previously investigated using a circRNA array analysis (Guo et al., 2018). The results indicated that 128 circRNAs were upregulated and 161 circRNAs were downregulated in pancreatic cancer tissues compared to adjacent normal tissues. Some differentially expressed circRNAs (e.g., circRNA_000780, circRNA_100435, circRNA_101252 and circRNA_103076) might be involved in pancreatic cancer progression by acting as miRNA sponges.

Given their close association with cancer biology, exploring deregulated circRNAs and their roles in cancer has been a research hotspot. In recent years, an expanding number of dysregulated circRNAs have been gradually identified in GI cancer, but their detailed functions and mechanisms in the development and progression of GI cancer are still equivocal. Further studies are needed to fully elucidate the regulatory mechanisms and clinical significance of circRNAs in GI cancer. In the case of circRNAs that are upregulated in GI cancer, depletion of oncogenic circRNAs might be achieved by RNA interference. For circRNAs that are downregulated in GI cancer, ectopic expression of tumor-suppressive circRNAs may represent a potential treatment option. Illumination of the therapeutic potential of circRNA-based approaches against cancer has become an important area of biomedical research. One of the central issues with circRNA-based therapies is discovering the optimal method to efficiently transport small interfering RNAs (siRNAs) or artificial circRNAs *in vivo*. Lipid nanoparticles represent an appropriate delivery system that protects siRNAs from degradation and facilitates their uptake by host cells (Tam et al., 2013). However, the efficiency of nanoparticle-mediated delivery of siRNAs or circRNA expression vectors is relatively low. Nanoparticle delivery systems must be optimized to overcome this limitation. Moreover, exosomes have exhibited great promise as transport vehicles for circRNA-targeting siRNAs or circRNA expression vectors (Ha et al., 2016). Exosomes appear to be superior drug carriers compared to synthetic nanoparticles. Nevertheless, exosomes have the challenges of the manufacturing scale and homogeneity. Studies aiming to perform an in-depth characterization of the factors and molecular pathways responsible for circRNA biogenesis will be helpful for the development of advanced delivery systems. Limited preclinical reports concerning the utility of circRNAs for treating cancer are available. Much more work is needed to validate the safety and effectiveness of circRNA-based therapeutics before their clinical use.

## 3 Circular RNAs and Gastrointestinal Cancer Chemoresistance

**FIGURE 2:**
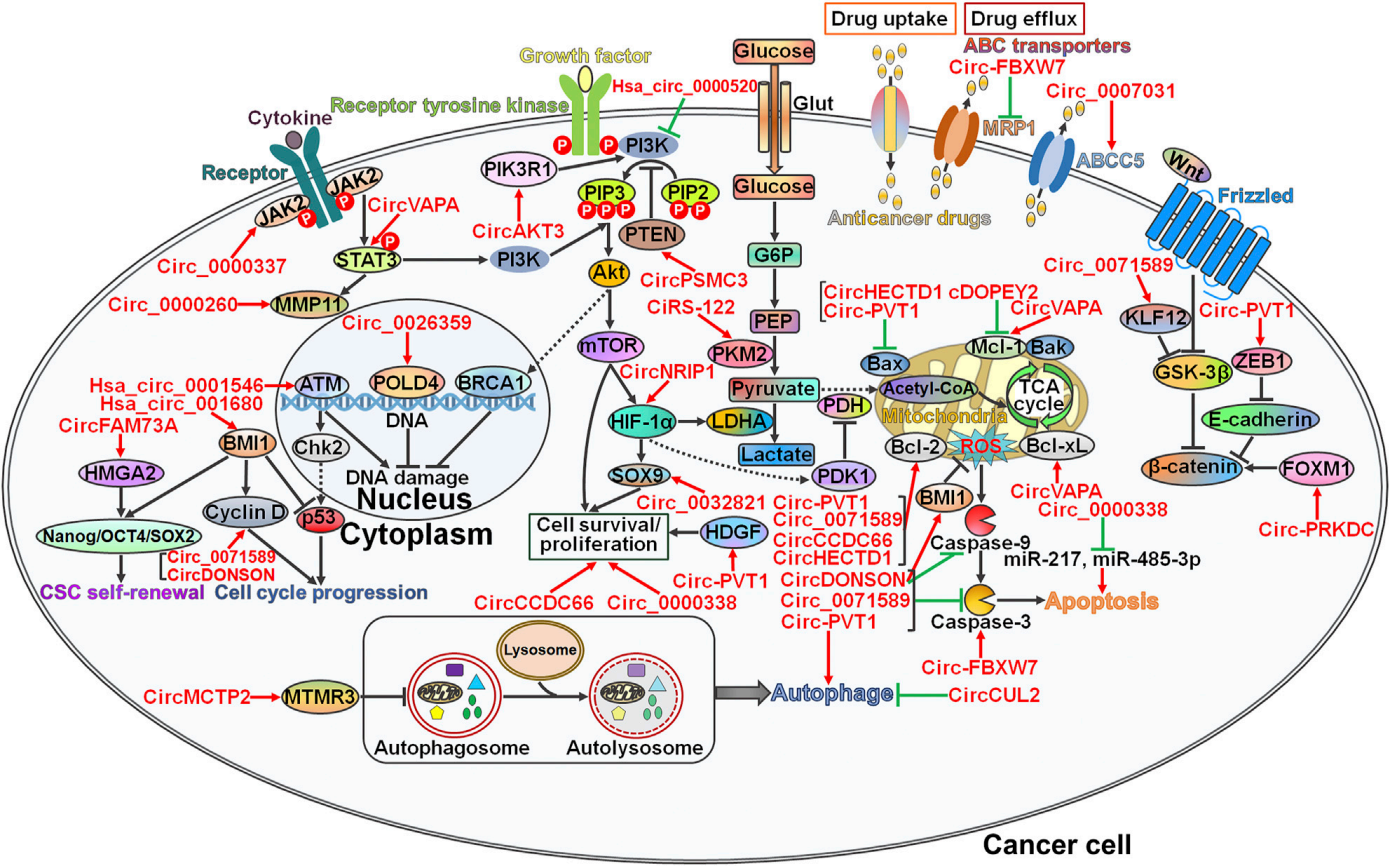
The underlying mechanisms involved in circRNA-mediated chemoresistance in gastrointestinal cancer. CircRNAs function as master regulators of drug resistance in gastrointestinal (GI) cancer via diverse pathways. Circ_0000337 and circVAPA strengthen chemotherapy-resistant characteristics of GI cancer by activating the JAK2/STAT3 signaling pathway. Circ_0000260 induces an increase in the expression level of MMP11, the downstream molecule of the JAK2/STAT3 pathway. As a result, circ_0000260 promotes cancer cell metastasis and confers chemoresistance to GI cancer cells. Circ_0026359 and hsa_circ_0001546 exert opposite effects on the DNA damage repair pathway, and they serve as important players in GI cancer chemoresistance. Circ_0071589 and circDONSON accelerate cell cycle progression by elevating Cyclin D expression. Consequently, these circRNAs induce the drug-resistant phenotype in GI cancer. CircFAM73A causes CSC self-renewal in GI cancer via upregulating stemness-relevant transcription factors (e.g., Nanog, OCT4 and SOX2) by reinforcing HMGA2 expression. Hsa_circ_001680 promotes CSC growth by inducing the key regulator of CSC self-renewal, BMI1, leading to the acquisition of chemoresistance in GI cancer. CircAKT3 and hsa_circ_0000520 affect the responsiveness of GI cancer cells to chemotherapy by regulating the PI3K/Akt signaling pathway. CircPSMC3 reduces the proliferation of GI cancer cells by increasing PTEN expression and sensitizes cancer cells to chemotherapeutic agents. Circ_0032821, circ-PVT1, circCCDC66 and circ_0000338 facilitate the survival and proliferation of GI cancer cells by controlling the expression of their cellular targets (e.g., SOX9 and HDGF). CiRS-122 and circNRIP1 favor glucose metabolism to fuel cancer cell growth, resulting in the development of drug resistance in GI cancer. Circ-FBXW7 decreases drug efflux transporter MRP1 expression to augment the cytotoxicity of anticancer drugs toward GI cancer cells. In contrast, circ_0007031 raises the expression level of ABCC5 and thus protects GI cancer cells from chemotherapeutic agent-induced cell killing. Various circRNAs have been involved in cell death pathways (apoptosis and autophagy). Particularly, circHECTD1 and circ-PVT1 diminish the expression of pro-apoptotic Bax. cDOPEY2, circVAPA, circ-PVT1, circ_0071589, circCCDC66 and circHECTD1 can target anti-apoptotic proteins such as Mcl-1, Bcl-2 and Bcl-xL. CircDONSON, circ_0071589 and circ-PVT1 limit the expression of the apoptotic effector caspase-3, while circ-FBXW7 has an antagonistic role. Circ_0000338 suppresses cell apoptosis by sponging miR-217 and miR-485-3p. Both circMCTP2 and circCUL2 block the autophagic flux. Given the association between autophagy and drug resistance, these two circRNAs play a critical role in regulation of chemotherapy response in GI cancer. Circ-PVT1 and circ-PRKDC mobilize the Wnt/β-catenin signaling cascade to drive the EMT process and cancer cell invasion. The EMT-inducing potential of circRNAs accounts for their promotive effects on the development of drug resistance in GI cancer. Arrows in red represent activation effect, and the ‘T’ symbols in green represent inhibition effect. JAK2, Janus kinase 2; STAT3, signal transducer and activator of transcription 3; MMP11, matrix metalloproteinase 11; HMGA2, high mobility group A2; Oct4, octamer-binding transcription factor 4; SOX2, SRY-box transcription factor 2; CSC, cancer stem cell; BMI1, B lymphoma Mo-MLV insertion region 1; MTMR3, myotubularin-related protein 3; ATM, Ataxia telangiectasia mutated; Chk2, checkpoint kinase 2; POLD4, DNA polymerase δ subunit 4; BRCA1, breast cancer type 1 susceptibility protein; PIK3R1, phosphatidylinositol 3-kinase regulatory subunit 1; PI3K, phosphatidylinositol 3-kinase; PIP3, phosphatidylinositol 3,4,5-trisphosphate; PIP2, phosphatidylinositol 4,5-bisphosphate; PTEN, phosphatase and tensin homolog; Akt, protein kinase B; mTOR, mammalian target of rapamycin; HIF-1α, hypoxia-inducible factor-1α; SOX9, SRY-box transcription factor 9; Glut, glucose transporter; G6P, glucose-6-phosphate; PEP, phosphoenolpyruvate; PKM2, the M2 isoform of pyruvate kinase; LDHA, lactate dehydrogenase A; HDGF, hepatoma-derived growth factor; PDH, pyruvate dehydrogenase; PDK1, pyruvate dehydrogenase kinase 1; MRP1, multidrug resistance protein 1; ABCC5, ATP-binding cassette subfamily C member 5; Bcl-2, B-cell lymphoma-2; Bax, Bcl-2-associated X protein; Mcl-1, myeloid cell leukemia-1; Bak, Bcl-2 antagonistic killer; TCA, tricarboxylic acid; ROS, reactive oxygen species; Bcl-xL, B-cell lymphoma-extra large; KLF12, kruppel-like factor 12; GSK-3β, glycogen synthase kinase-3β; ZEB1, Zinc finger E-box-binding homeobox 1; FOXM1, forkhead fox protein M1.

Drug resistance is still a major clinical challenge in effective cancer therapy. The development of drug resistance in cancer involves diverse mechanisms, including DNA damage repair, modulation of cell viability and proliferation, manipulation of cell death-relevant pathways, regulation of glucose metabolism, induction of the cancer stem cell (CSC) phenotype, and modification of drug efflux and metabolism (Micallef and Baron, 2021). CircRNAs play an important role in regulating the chemoresistance of GI cancer by interfering with these pathways (Figure 2).

### 3.1 DNA Damage Repair Pathway

CRC is ranked as the fourth leading cause of cancer-related death worldwide (Ren et al., 2021). CRC is commonly diagnosed at an advanced stage, and chemotherapy represents an important treatment option for patients with advanced CRC. CircRNAs have been shown to affect the sensitivity of CRC cells to different therapeutic drugs, such as 5-FU, OXA and irinotecan. In a recent report, Yao F. et al. (2021) detected circRNA expression profiles and identified differentially expressed circRNAs in 5-FU- and cisplatin (DDP)-resistant CRC cells compared with parental CRC cells by performing RNA-seq and bioinformatics analyses. The results showed that 7,393 and 7,385 circRNAs were expressed in 5-FU- and DDP-resistant CRC cells, respectively. In addition, 48 and 90 differentially expressed circRNAs were identified among the discovered circRNAs in 5-FU- and DDP-resistant CRC cells compared with sensitive CRC cells. Importantly, differentially expressed circRNAs might be associated with drug resistance-relevant pathways, including the DNA repair pathway and the Hippo signal transduction cascade. *In vitro* experimental results verified that hsa_circ_002482 upregulation markedly increased the chemosensitivity of CRC cells (Table 1). Paradoxically, hsa_circ_002482 reduced the expression of drug-sensitizing miR-503-5p (Yang et al., 2017). It was unclear whether miR-503-5p participated in hsa_circ_002482-regulated drug susceptibility. Considerable research efforts should be devoted to fully elucidating the definite mechanisms by which hsa_circ_002482 altered CRC chemoresistance. Existing evidence suggested that the impact of circRNAs on CRC drug resistance was partially attributable to the regulation of the DNA repair pathway. Additional investigation is still needed to define the interaction between chemoresistance-associated circRNAs and the DNA repair pathway in CRC.

**TABLE 1:** Chemoresistance-relevant circRNAs in gastrointestinal cancer.

Cancer	CircRNA	Drugs	Expression	Targets	Function	References
Colorectal cancer	Hsa_circ_002482	5-Fluorouracil, cisplatin	Downregulation	miR-503-5p	Sensitivity	Yao et al. (2021a)
Gastric cancer	Circ_0026359	Cisplatin	Upregulation	miR-1200/POLD4	Resistance	Zhang et al. (2020b)
Gastric cancer	CircAKT3	Cisplatin	Upregulation	miR-198/PIK3R1	Resistance	Huang et al. (2019b)
Gastric cancer	Hsa_circ_0001546	Oxaliplatin	Downregulation	miR-421/ATM/Chk2/p53	Sensitivity	Wu et al. (2020)
Colorectal cancer	CircCCDC66	Oxaliplatin	Upregulation	Cell proliferation/survival-associated genes	Resistance	Lin et al. (2020)
Gastric cancer	Circ-PVT1	Cisplatin	Upregulation	miR-152-3p/HDGF, Bax, cleaved caspase-3, Bcl-2	Resistance	Wang et al. (2021e)
Esophageal squamous cell carcinoma	CircPSMC3	Gefitinib	Downregulation	miR-10a-5p/PTEN	Sensitivity	Zhu et al. (2021)
Colorectal cancer	Circ_0000338	5-Fluorouracil	Upregulation	miR-217, miR-485-3p	Resistance	Zhao et al. (2021)
Colorectal cancer	Circ_0071589	Cisplatin	Upregulation	miR-526b-3p/KLF12, Cyclin D1, Bcl-2, cleaved caspase-3	Resistance	Zhang et al. (2021b)
Gastric cancer	CircVAPA	Cisplatin	Upregulation	miR-125b-5p/STAT3, Bcl-xL, Mcl-1, Survivin	Resistance	Deng et al. (2021)
Gastric cancer	CircDONSON	Cisplatin	Upregulation	miR-802/BMI1, cleaved caspase-3/-9, p27, Cyclin D1	Resistance	Liu et al. (2020b)
Gastric cancer	Hsa_circ_0000520	Herceptin	Downregulation	PI3K/Akt, Bax, Bcl-2	Sensitivity	Lv et al. (2020)
Gastric cancer	CircCCDC66	Cisplatin	Upregulation	miR-618/Bcl-2	Resistance	Zhang et al. (2020a)
Gastric cancer	CircHECTD1	Diosbulbin-B	Upregulation	miR-137/PBX3, Bax, Bcl-2	Resistance	Lu et al. (2021)
Gastric cancer	CircMCTP2	Cisplatin	Downregulation	miR-99a-5p/MTMR3, P62, LC3-II	Sensitivity	Sun et al. (2020a)
Gastric cancer	CircCUL2	Cisplatin	Downregulation	miR-142-3p/ROCK2, P62, Beclin 1, LC3	Sensitivity	Peng et al. (2020)
Esophageal squamous cell carcinoma	cDOPEY2	Cisplatin	Downregulation	CPEB4, Mcl-1	Sensitivity	Liu et al. (2021)
Gastric cancer	CircNRIP1	5-Fluorouracil	Upregulation	miR-138-5p/HIF-1α	Resistance	Xu et al. (2020)
Colorectal cancer	Hsa_circ_001680	Irinotecan	Upregulation	miR-340/BMI1	Resistance	Jian et al. (2020)
Gastric cancer	CircFAM73A	Cisplatin	Upregulation	miR-490-3p/HMGA2, Nanog, OCT4, SOX2, β-catenin	Resistance	Xia et al. (2021)
Colorectal cancer	Circ-PRKDC	5-Fluorouracil	Upregulation	miR-375/FOXM1	Resistance	Chen et al. (2020)
Gastric cancer	Circ-PVT1	Paclitaxel	Upregulation	miR-124-3p/ZEB1	Resistance	Liu et al. (2019)
Gastric cancer	Circ_ASAP2	Cisplatin	Upregulation	miR-330-3p/NT5E	Resistance	Sun et al. (2021)
Gastric cancer	Circ_0000260	Cisplatin	Upregulation	miR-129-5p/MMP11	Resistance	Liu et al. (2020a)
Esophageal squamous cell carcinoma	Circ_0006168	Paclitaxel	Upregulation	miR-194-5p/JMJD1C	Resistance	Qu et al. (2021)
Colorectal cancer	Circ_0007031	5-Fluorouracil	Upregulation	miR-133b/ABCC5	Resistance	He et al. (2020)
Colorectal cancer	Circ_0000338	5-Fluorouracil	Upregulation	Cell viability	Resistance	Hon et al. (2019)
Colorectal cancer	CiRS-122	Oxaliplatin	Upregulation	miR-122/PKM2	Resistance	Wang et al. (2020c)
Colorectal cancer	Circ-FBXW7	Oxaliplatin	Downregulation	miR-18b-5p, MRP1, Mcl-1, cleaved caspase-3	Sensitivity	Xu et al. (2021)
Gastric cancer	Circ_0032821	Oxaliplatin	Upregulation	miR-515-5p/SOX9	Resistance	Zhong et al. (2021)
Gastric cancer	Circ-PVT1	Cisplatin	Upregulation	miR-30a-5p/YAP1, LC3-II/I, P-gp, P62	Resistance	Yao et al. (2021b)
Esophageal squamous cell carcinoma	Circ_0000337	Cisplatin	Upregulation	miR-337-3p/JAK2	Resistance	Zang et al. (2021)

GC is the second most common cause of cancer-associated mortality worldwide (Bray et al., 2018). The main GC treatment remains cytotoxic chemotherapy. DDP is a commonly used chemotherapeutic agent in clinical GC treatment. Nevertheless, DDP resistance has become a serious obstacle undermining its therapeutic efficacy. CircRNAs are critical regulators of GC chemoresistance, suggesting their clinical implications as novel therapeutic targets for GC (Wei et al., 2020). The linkage between circRNAs and DDP resistance has been gradually disclosed, providing novel insights into the mechanisms associated with GC chemoresistance. Circ_0026359 was shown to be overexpressed in DDP-resistant GC compared with sensitive tissues (Zhang Z. et al., 2020). Circ_0026359 absence increased miR-1200 activity and thus lowered the expression of DNA polymerase δ subunit 4 (POLD4). POLD4 downregulation caused DNA fragmentation and genomic instability in DDP-resistant GC cells, thus reducing cell viability and promoting apoptosis and DDP sensitivity in GC cells. The miR-1200/POLD4 pathway mediated the function of circ_0026359 in the DDP resistance of GC. The expression of circAKT3 was overtly upregulated in DDP-resistant GC tissues and cells compared to sensitive samples (Huang et al., 2019b). Mechanistically, circAKT3 functioned as a miR-198 sponge to upregulate phosphatidylinositol 3-kinase regulatory subunit 1 (PIK3R1), which activated the phosphatidylinositol 3-kinase (PI3K)/protein kinase B (Akt) signaling cascade. Activation of the PI3K/Akt pathway caused the upregulation of breast cancer type 1 susceptibility protein (BRCA1), thereby inducing DNA damage repair and contributing to increased DDP resistance in GC cells. These findings highlighted a novel therapeutic option for DDP-resistant GC. The exact mechanism by which BRCA1 modulates DNA damage repair in GC remains to be elucidated. In contrast, hsa_circ_0001546 was apparently downregulated in GC tissues compared with adjacent normal tissues (Wu et al., 2020). Hsa_circ_0001546 increased the expression of the DNA damage-inducible kinase ataxia telangiectasia mutated (ATM) by absorbing miR-421. ATM/checkpoint kinase 2 (Chk2)/p53 signaling played a pivotal role in the DNA damage response, cell apoptosis and drug resistance in cancer (Shi et al., 2012; Yao et al., 2017). As a result, hsa_circ_0001546 suppressed cell proliferation and sensitized OXA-resistant GC cells to OXA by activating the ATM/Chk2/p53 signaling pathway. Based on these data, hsa_circ_0001546 might represent a therapeutic target for the treatment of OXA-resistant GC. Collectively, circRNAs could stimulate or suppress the DNA damage repair pathway, leading to altered sensitivity of GC cells to chemotherapy.

Chemotherapeutic drugs may induce cancer cell death by triggering DNA damage. Cancer cells activate DNA damage repair pathways to resist chemotherapeutic agents. Numerous genes and signaling pathways are involved in DNA damage repair. Alterations in their expression levels or activities significantly affect the responsiveness of cancer cells to chemotherapy and cancer progression. Multiple lines of evidence have highlighted the emerging roles of circRNAs in GI cancer chemoresistance through the regulation of DNA damage-responsive genes or signaling cascades. Targeting circRNAs that modify DNA damage repair pathways may be a potential therapeutic option to address drug resistance in GI cancer. DNA damage repair is mainly triggered by PI3K-related kinase (PIKK) family proteins including ATM, ATM- and Rad3-related (ATR) kinase and DNA-dependent protein kinase catalytic subunit (DNA-PKcs), followed by the activation of downstream reactions (Lukas et al., 2011). The regulatory function of circRNAs in the ATR and DNA-PKcs pathways is yet to be determined. The interplay between circRNAs and DNA damage repair pathways is worthy of further study. Current studies predominantly focus on the roles of circRNAs in nuclear DNA damage repair pathways in GI cancer. The involvement of circRNAs in the mitochondrial DNA (mtDNA) repair pathway remains unknown. Inhibition of the mtDNA repair pathway may diminish unfavorable effects on normal cells and shows promise as a more effective anticancer therapy. Future studies are warranted to characterize circRNA-associated regulatory networks implicated in the mtDNA repair pathway.

### 3.2 Cell Viability and Proliferation

DExH-box helicase 9 (DHX9) functions as a critical regulator of circRNA biogenesis and modulates the base-pairing interaction of intronic sequences bracketing the circularized region (Aktas et al., 2017). OXA exposure enhanced circCCDC66 expression through PI3KK-mediated DHX9 phosphorylation in CRC cells (Lin et al., 2020). CircCCDC66 knockdown evidently reduced the survival of OXA-resistant CRC cells, thereby blocking the development of chemoresistance. A genome-wide analysis of circCCDC66 knockdown-resistant cells and control resistant cells using RNA-seq revealed that circCCDC66 modulated the expression of various genes associated with cell proliferation and survival by competitively binding multiple miRNAs. The upregulation of circCCDC66 might confer a survival advantage to OXA-resistant CRC cells in response to OXA treatment. Nevertheless, the signaling pathways implicated in circCCDC66-induced chemoresistance are worthy of continued exploration. It seems that circRNAs play crucial roles in fine-tuning the expression of genes associated with cell proliferation by sponging diverse miRNAs. The role of circRNA/miRNA/mRNA interaction networks in GI cancer cell proliferation and chemoresistance needs to be systematically studied.

Circ-PVT1 was apparently upregulated in DDP-resistant GC tissues and cells compared to chemosensitive samples (Wang et al., 2021e). Downregulation of circ-PVT1 reduced the resistance of GC cells to DDP, thereby attenuating the malignant features of DDP-resistant GC cells. Mechanistically, circ-PVT1 was able to directly target miR-152-3p, which specifically decreased the expression of hepatoma-derived growth factor (HDGF). HDGF functions as an oncogene with abnormally elevated activity in various cancers (Chu et al., 2019). Circ-PVT1 interference promoted the apoptosis of DDP-resistant GC cells by increasing the levels of Bcl-2-associated X protein (Bax) and cleaved caspase-3 and decreasing the level of Bcl-2. Upregulation of miR-152-3p or knockdown of HDGF suppressed the chemoresistance and malignancy of DDP-resistant GC cells, whereas these effects were reversed by circ-PVT1 overexpression. Thus, circ-PVT1 controlled GC chemoresistance via the miR-152-3p/HDGF pathway. Targeting circ-PVT1 in GC may be an attractive treatment strategy.

EC has the sixth highest mortality rate among all malignancies worldwide and can be divided into two major histological types, ESCC and esophageal adenocarcinoma (EAC) (Uhlenhopp et al., 2020). The expression level of circPSMC3 was declined in gefitinib-resistant ESCC cells compared to sensitive cells (Zhu et al., 2021). Upregulation of circPSMC3 improved the sensitivity of ESCC cells to gefitinib. The functional downstream pathway of circPSMC3 was also identified. CircPSMC3 elevated the expression of phosphatase and tensin homolog (PTEN) by lowering miR-10a-5p levels. Restoration of miR-10a-5p expression or knockdown of PTEN significantly counteracted the effect of circPSMC3 overexpression on increasing gefitinib sensitivity in ESCC. Thus, the circPSMC3/miR-10a-5p/PTEN axis might constitute the molecular mechanisms involved in ESCC chemoresistance. PTEN is a well-characterized tumor suppressor with a crucial role in modulating the anti-apoptotic and survival pathways (McLoughlin et al., 2018). Inhibition of PTEN-targeting oncogenic miRNAs is of prime interest as an anticancer treatment. CircPSMC3 post-transcriptionally coordinated PTEN expression via acting as a miRNA sponge. Reactivation of circPSMC3 may potentially be beneficial for the treatment of chemoresistant ESCC.

Uncontrolled cell proliferation plays a crucial role in cancer progression. Many anticancer agents, including alkylating drugs (e.g., DDP and OXA), antimetabolic drugs (e.g., 5-FU) and DNA crosslinking drugs (e.g., DDP and mitomycin C), suppress cancer cell proliferation. Chemoresistant cancer cells can tolerate anticancer drugs to a certain extent. CircRNAs are capable of regulating drug resistance in GI cancer by targeting important genes involved in cell proliferation pathways. Various miRNAs and proteins have been identified as key participants in cancer cell proliferation (Zhang N. et al., 2021). CircRNAs may alter cell proliferation and drug sensitivity in GI cancer by interacting with these miRNAs and proteins. The intertwined competitive endogenous RNA (ceRNA) regulatory axes underlying GI cancer chemoresistance require further study.

### 3.3 Cell Death Pathways

In contrast to 5-FU-sensitive tissues, circ_0000338 was upregulated in 5-FU-resistant CRC tissues (Zhao et al., 2021). Depletion of circ_0000338 sensitized 5-FU-resistant CRC cells to 5-FU by promoting the apoptosis and inhibiting the proliferation of CRC cells. Remarkably, circ_0000338 acted as a ceRNA to soak up miR-217 and miR-485-3p. Silencing of miR-217 or miR-485-3p attenuated circ_0000338 knockdown-mediated increase in the chemosensitivity of 5-FU-resistant CRC cells. The miRNA sponging activity was crucial for the effect of circ_0000338 on 5-FU resistance in CRC. However, downstream targets of these two miRNAs were not identified in this study. Thus, further functional and mechanistic studies are required to define the function of circ_0000338-miR-217/miR-485-3p feedback loops in CRC chemoresistance. Similarly, circ_0071589 expression was significantly elevated in DDP-resistant CRC tissues compared with sensitive tissues (Zhang W. et al., 2021). Circ_0071589 increased the expression of the oncogene kruppel-like factor 12 (KLF12) by interacting with miR-526b-3p. Moreover, downregulation of circ_0071589 inhibited the proliferation and promoted the apoptosis of DDP-resistant CRC cells by downregulating Cyclin D1 and Bcl-2 and upregulating cleaved caspase-3. These effects contributed to the suppression of chemoresistance in DDP-resistant CRC cells. Silencing of miR-526b-3p abolished the effect of circ_0071589 knockdown on DDP resistance and cancer malignancy in DDP-resistant CRC cells. In addition, circ_0071589 interference aggravated DDP-induced tumor inhibition in a murine xenograft model. Accordingly, circ_0071589 was proposed to impair the intrinsic apoptotic pathway and might serve as a novel target for improving the efficacy of chemotherapy in CRC.

CircVAPA was expressed at high levels in GC tissues, and its downregulation enhanced the susceptibility of chemoresistant GC cells to DDP (Deng et al., 2021). CircVAPA upregulated STAT3 by interacting with miR-125b-5p. Consistently, STAT3 downstream proteins, including B-cell lymphoma-extra large (Bcl-xL), myeloid cell leukemia-1 (Mcl-1) and Survivin, were upregulated in DDP-resistant GC cells. As a result, circVAPA promoted the proliferation and inhibited the apoptosis of GC cells. The miR-125b-5p inhibitor or STAT3 upregulation reversed circVAPA knockdown-induced chemosensitivity in GC cells. It was thus proposed that the miR-125b-5p/STAT3 axis mediated the regulatory effects of circVAPA on GC chemoresistance. Another upregulated circRNA in DDP-resistant GC tissues and cells, circDONSON, acted as a molecular sponge by competing for miR-802 binding to affect the expression of its target, B lymphoma Mo-MLV insertion region 1 (BMI1) (Liu Y. et al., 2020). Functionally, circDONSON knockdown sensitized GC cells to DDP via the miR-802/BMI1 axis *in vitro* and *in vivo*. CircDONSON deficiency elevated the levels of cleaved caspase-3, cleaved caspase-9 and p27, while reducing the level of Cyclin D1 in DDP-resistant GC cells. Thus, circDONSON downregulation reduced cell viability and accelerated cell apoptosis in DDP-resistant cells. These results suggested the important role of circDONSON in GC, indicating that it represented a promising therapeutic target for improving chemotherapy effectiveness in GC patients. In contrast, hsa_circ_0000520 was downregulated in Herceptin-resistant GC cells compared with GC cells (Lv et al., 2020). Hsa_circ_0000520 overexpression apparently decreased the viability and promoted the apoptosis of chemoresistant GC cells by upregulating Bax and downregulating Bcl-2. Hsa_circ_0000520-mediated inactivation of the PI3K/Akt signaling pathway contributed to its anticancer activity. Conversely, induction of the PI3K/Akt pathway eliminated the inhibitory effect of hsa_circ_0000520 on Herceptin resistance in GC. Altogether, hsa_circ_0000520 enhanced Herceptin sensitivity in GC cells by blocking the PI3K/Akt signaling cascade. CircCCDC66 was upregulated in DDP-resistant GC tissues and cells compared to sensitive samples (Zhang Q. et al., 2020). *In vitro* and *in vivo* experiments indicated that circCCDC66 suppressed GC cell apoptosis and induced DDP resistance in GC cells by targeting miR-618 to facilitate Bcl-2 release. CircHECTD1 was overexpressed in GC tissues in contrast with adjacent normal tissues (Lu et al., 2021). CircHECTD1 deletion expedited the apoptosis of GC cells by upregulating Bax and downregulating Bcl-2. CircHECTD1 increased the expression of pre-B-cell leukemia transcription factor 3 (PBX3) by sponging miR-137. PBX3 is a cancer-relevant protein that has been reported to be associated with cancer cell metastasis (Han et al., 2014). CircHECTD1 absence reinforced diosbulbin-B (DB) sensitivity in GC cells, and this effect was alleviated by a miR-137 inhibitor. CircHECTD1 altered cell viability, apoptosis and drug resistance in DB-induced GC cells, which provided theoretical support for its application as a promising therapeutic target for GC. The key mediators of the intrinsic apoptotic pathway participate in carcinogenesis and chemotherapy resistance and can be targeted for anticancer therapeutic approaches. The multi-targeted strategies may be more efficacious to combat drug resistance in cancer. CircRNAs have the ability to target diverse apoptosis-associated proteins. Substitution of pro-apoptotic circRNAs or inhibition of anti-apoptotic circRNAs could be used to design and develop multi-target-based anticancer strategies for the treatment of GI cancer.

CircMCTP2 was downregulated in DDP-resistant GC tissues and cells (Sun G. et al., 2020). CircMCTP2 inhibited autophagic cell death in DDP-resistant cells by modulating P62 and LC3-II levels. Mechanistically, circMCTP2 increased the level of the autophagy inhibitor myotubularin-related protein 3 (MTMR3) by interacting with miR-99a-5p. CircMCTP2 sensitized GC cells to DDP through suppressing autophagy by restoration of MTMR3 expression. Another downregulated circRNA, circCUL2, induced the upregulation of rho-associated coiled-coil-containing protein kinase-2 (ROCK2) by sponging miR-142-3p (Peng et al., 2020). CircCUL2 inhibited autophagy by regulating miR-142-3p, as reflected by the altered expression of autophagy-related markers, including P62, Beclin 1 and LC3. Consequently, circCUL2 enhanced DDP sensitivity in GC cells via miR-142-3p/ROCK2-mediated autophagy. Particularly, circCUL2 restricted protective autophagy to increase the chemosensitivity of GC cells. In some cases, autophagy performs an anti-carcinogenic function. The roles of circRNAs in autophagy regulation deserve further attention. Specifically, it is important to discern whether circRNA-mediated autophagy is beneficial or harmful. Additional efforts are required to investigate how circRNAs regulate varied forms of autophagy. The mechanisms by which circRNA-regulated autophagy governs cancer development and chemoresistance have not yet been fully elucidated. Further work is needed to understand the crosstalk between circRNA-associated ceRNA networks and the autophagic flux in chemoresistant GI cancer.

Hsa_circ_0008078, also named cDOPEY2, was apparently downregulated in DDP-resistant ESCC cells compared with chemosensitive cells (Liu et al., 2021). cDOPEY2 upregulation markedly strengthened the cytotoxicity of DDP toward DDP-resistant ESCC cells by promoting cell apoptosis, as reflected by the downregulation of the anti-apoptotic protein Mcl-1. Furthermore, cDOPEY2 served as a scaffolding molecule to facilitate the interaction between cytoplasmic polyadenylation element-binding protein 4 (CPEB4) and the E3 ligase tripartite motif-containing protein 25 (TRIM25), leading to the ubiquitination and degradation of CPEB4. The binding of CPEB4 to the *Mcl-1* mRNA enhanced the expression of Mcl-1. cDOPEY2-mediated degradation of CPEB4 abrogated this effect. Gain-of-function experiments showed that the restoration of cDOPEY2 expression alleviated DDP resistance in ESCC by repressing CPEB4-mediated Mcl-1 translation. This study established a previously uncharacterized mechanism underpinning a critical role of circRNAs in altering cancer chemoresistance.

Apoptosis and autophagy are closely associated with carcinogenesis. The crosstalk between apoptosis and autophagy has been characterized (Xie et al., 2020). Apoptosis represents one of the predominant ways of cancer cell death. Autophagy exerts opposite effects on cancer pathogenesis. Autophagy can initiate cancer cell apoptosis in combination with anticancer drugs. In some circumstances, autophagy suppresses chemotherapeutic agent-induced apoptosis in cancer. Autophagy constitutes a major factor contributing to cancer drug resistance. Studies revealing the interplay between apoptosis and autophagy facilitate improvements in the therapeutic efficacy of anticancer agents against cancer. The cellular apoptosis and autophagy pathways may be modulated by shared regulatory factors and signal transduction cascades, including ncRNAs, p53 and the PI3K/Akt/mammalian target of rapamycin (mTOR) pathway. CircRNAs have emerged as upstream effectors of cell death pathways in GI cancer. CircRNAs possess the ability to tip the balance between cell death signalings and hence may improve the efficacy of anticancer medications. It is unclear whether circRNAs simultaneously regulate the apoptotic and autophagic pathways in GI cancer by controlling the same factor or signaling pathway. Crosstalk between ncRNAs affecting apoptosis and autophagy may exist. Thus, the impact of circRNAs on the interaction pattern between apoptosis and autophagy in GI cancer necessitates further exploration, which will provide a basis for the development of more effective therapeutic strategies to combat chemoresistant GI cancer.

### 3.4 Glycolysis

Hypoxia-induced chemoresistance has been considered a major hurdle to the development of successful therapy for GC (Karakashev and Reginato, 2015). Xu et al. (Xu et al., 2020) found that circNRIP1 was expressed at high levels in hypoxic GC cells and promoted hypoxia-induced 5-FU resistance in GC cells. Mechanistically, circNRIP1 acted as a miR-138-5p sponge and modulated its target, hypoxia-inducible factor-1α (HIF-1α). Glycolysis is the predominant energy-generating pathway in hypoxic cancer cells, leading to the development of hypoxia-induced chemoresistance and the survival of cancer cells under hypoxic conditions (Tavares-Valente et al., 2013; Bhattacharya et al., 2014). HIF-1α plays a key role in modulating glycolysis and is involved in cancer drug resistance (Shukla et al., 2017). Thus, HIF-1α-dependent glucose metabolism might contribute to hypoxia-induced chemoresistance in GC. Consistent with this hypothesis, pharmacological blockade of glycolysis reversed the effects of circNRIP1 on hypoxia-induced 5-FU resistance in GC cells, suggesting that circNRIP1 had emerged as an important regulator of hypoxia-induced chemoresistance via HIF-1α-mediated glucose metabolism in GC by targeting miR-138-5p (Xu et al., 2020).

Hypoxia is a common microenvironmental feature in solid tumors (Masoud and Li, 2015). Cancer cells can activate the transcription factor HIF-1α under hypoxic conditions. HIF-1α mediates metabolic switching by upregulating glucose transporters and glycolytic enzymes, favoring the adaptation of cancer cells to hypoxia and eventually inducing drug resistance (Zheng et al., 2021). Glycolysis meets the energetic demands for cellular functions and establishes biological blocks for cancer cells, leading to the rapid growth of cancer cells (Beltran-Anaya et al., 2016). A number of enzymes are implicated in glucose metabolism, such as hexokinase (HK), pyruvate kinase (PK) and lactate dehydrogenase (LDH). Targeting glycolytic enzymes with ncRNAs would add to the complexity of the glucose metabolism process in hypoxic cancer cells. Existing evidence has suggested the effect of the circRNA/miRNA/HIF-1α regulatory axis on hypoxia-induced drug resistance in GC. The expression and activity of the downstream genes responsible for catalyzing glucose metabolism remain to be validated. In addition, the direct impact of circRNAs on glycolytic enzymes merits additional research.

### 3.5 Self-Renewal of Cancer Stem Cells

CSCs, also known as tumor-initiating cells (TICs), are a unique subpopulation of self-renewing cells with high carcinogenic potential and higher resistance to conventional therapies than other cells within a tumor (Najafi et al., 2019). Due to their self-renewing property and ability to differentiate into heterogeneous lineages of cancer cells, CSCs are responsible for tumor progression and recurrence (Phi et al., 2018). CSC-mediated chemoresistance can be partially explained by their quiescence or dormancy, increased drug efflux and avoidance of harmful stresses (Li et al., 2021). Recent evidence suggests that circRNAs are critical regulators of CSC growth that modify drug resistance in GI cancer. A higher expression level of hsa_circ_001680 was observed in CRC tissues than in matched adjacent normal tissues (Jian et al., 2020). *In vitro* and *in vivo* evidence showed that hsa_circ_001680 overexpression promoted the proliferation and migration of CRC cells. BMI1 is a critical transcription factor required for the maintenance and self-renewal of CSCs (Wang et al., 2016). Hsa_circ_001680 enhanced the CSC population of CRC cells and thus contributed to irinotecan chemotherapy resistance in CRC cells by sponging miR-340 to increase BMI1 expression (Jian et al., 2020). Hsa_circ_001680 were able to control the growth of CSCs in CRC by inducing the key regulator of CSC self-renewal, which supported the maintenance of CSC stemness and chemoresistance.

CircFAM73A was apparently upregulated in GC, and its upregulation was strongly correlated with the poor prognosis of GC patients (Xia et al., 2021). CircFAM73A fostered GC cell proliferation, migration and DDP resistance. Moreover, circFAM73A boosted stem cell-like properties in GC cells by upregulating stemness-related transcription factors (e.g., Nanog, OCT4 and SOX2). A subsequent functional study indicated that circFAM73A coordinated the expression of high mobility group A2 (HMGA2) by sequestering miR-490-3p. *In vitro* and *in vivo* experimental results revealed that the positive effect of circFAM73A on GC cell self-renewal and malignancy was counteracted by HMGA2 depletion. Thus, circFAM73A regulated the CSC-like properties and malignant behaviors of GC by increasing HMGA2 expression. Additionally, circFAM73A bound to heterogeneous nuclear ribonucleoprotein K (HNRNPK) and facilitated β-catenin stabilization, hence aggrandizing the CSC-like properties of GC. CircFAM73A targeted stemness-related proteins through its miRNA sponging and protein binding activities, suggesting its broad implication in CSC self-renewal. Altogether, circFAM73A strengthened the self-renewal capability of GC cells, leading to DDP resistance and cancer progression.

CSCs are implicated in cancer initiation and development, as well as the acquisition of drug resistance. CSCs have been considered a promising therapeutic target for conquering cancer drug resistance. CircRNAs regulate the growth of CSCs in GI cancer by targeting stemness-related transcription factors and relevant miRNAs. Nevertheless, the multifaceted contributions of circRNAs to regulating CSC biology have not been fully delineated thus far. Additional studies should be conducted to elucidate the functional activity of circRNAs in modifying CSC features in GI cancer. Various cellular molecules and signal transduction cascades have been reported to be involved in the regulation of CSC functions, including Bcl-2, STAT3 and transforming growth factor-β (TGF-β) signaling (Kyriazi et al., 2020). The mechanisms by which CSCs facilitate cancer drug resistance include the induction of the epithelial-mesenchymal transition (EMT) program, upregulation of multidrug resistance (MDR) proteins, and regulation of the tumor environment (e.g., hypoxia and inflammation) (Phi et al., 2018). Ongoing studies are critical to explore whether circRNAs interfere with a cascade of signaling events contributing to CSC-mediated drug resistance and subsequent cellular effects in GI cancer. CSC-targeted therapeutic approaches have tremendous potential to completely eliminate cancer cells. CircRNAs have been identified as novel targets for affecting CSC properties in GI cancer. Therapies based on modulation of stemness-associated circRNAs may be feasible and effective in specifically eradicating progenitor cells and CSCs. Nevertheless, it should be noted that the tumor heterogeneity and ever-changing tumor microenvironment pose huge challenges for therapies targeting CSCs. Therefore, a detailed understanding of CSC characteristics and tumor microenvironment is a significant prerequisite for developing CSC-targeted therapeutic approaches against GI cancer.

### 3.6 Epithelial-Mesenchymal Transition, Invasion and Metastasis

The expression of circ-PRKDC was significantly upregulated in 5-FU-resistant CRC tissues and cells compared with sensitive CRC samples (Chen et al., 2020). Silencing of circ-PRKDC sensitized 5-FU-resistant CRC cells to 5-FU and inhibited cell invasion. Circ-PRKDC deficiency repressed the Wnt/β-catenin pathway by regulating the miR-375/forkhead fox protein M1 (FOXM1) axis. Deletion of miR-375 abolished the inhibitory effects of circ-PRKDC knockdown on CRC chemoresistance and cell invasion in 5-FU-resistant cells. Consequently, circ-PRKDC exerted a positive role in 5-FU resistance in CRC by orchestrating the miR-375/FOXM1 axis and the Wnt/β-catenin pathway. Zinc finger E-box-binding homeobox 1 (ZEB1) is an important transcriptional repressor of E-cadherin that accelerates the EMT program, migration and invasion in GC (Jia et al., 2012). Circ-PVT1 was overexpressed in paclitaxel (PTX)-resistant GC cells and increased ZEB1 expression by tethering miR-124-3p (Liu et al., 2019). Silencing of circ-PVT1 reduced PTX resistance, enhanced PTX-induced apoptosis and blocked the invasion of GC cells, while these effects were counteracted by miR-124-3p downregulation. Inhibition of ZEB1 improved the sensitivity of PTX-resistant GC cells to PTX. Approaches targeting circ-PVT1 might represent a promising therapeutic strategy for GC. The Wnt/β-catenin signaling cascade is considered critical to the activation of EMT in cancer. CircRNAs can hijack the Wnt/β-catenin pathway to regulate cancer cell invasion, metastasis and drug resistance by targeting key proteins and transcription factors involved in this pathway.

Circ_ASAP2 was expressed at high levels in DDP-resistant GC tissues and cells (Sun et al., 2021). Circ_ASAP2 silencing enhanced DDP sensitivity and apoptosis, and retarded the proliferation, migration and invasion of DDP-resistant GC cells. In terms of mechanism, circ_ASAP2 directly targeted miR-330-3p to upregulate the expression of ecto-5′-nucleotidase (NT5E), which is associated with tumor invasion and metastasis (Wang et al., 2008). Circ_ASAP2 enhanced DDP resistance and promoted the functional behaviors of resistant GC cells by targeting the miR-330-3p/NT5E axis. Circ_0000260 also showed higher expression levels in DDP-resistant GC tissues than in sensitive tumor tissues (Liu S. et al., 2020). Matrix metalloproteinase 11 (MMP11) functions as a key driver of cancer development and metastasis (Yang et al., 2019). *In vitro* and *in vivo* experimental studies verified that circ_0000260 knockdown reduced DDP resistance and impeded the malignancy of resistant GC cells by modulating the expression of miR-129-5p and its target MMP11. These results revealed a vital mechanism underlying the role of circ_0000260 in DDP resistance of GC. Circ_0006168 was upregulated in PTX-resistant ESCC tissues compared to oesophageal epithelial cells and sensitive ESCC cells (Qu et al., 2021). Circ_0006168 depletion enhanced the cytotoxicity of PTX toward resistant ESCC cells. It sponged miR-194-5p to upregulate jumonji domain containing 1C (JMJD1C). Downregulation of JMJD1C improved PTX sensitivity and suppressed the malignant behaviors of PTX-resistant ESCC cells. Circ_0006168 deficiency restrained tumor growth *in vivo* by increasing miR-194-5p expression and reducing JMJD1C expression. The specific physiological mechanisms underlying the roles of circ_0006168 in PTX resistance of ESCC require additional exploration. The clinical significance of circ_0006168 remains to be further validated. In some cases, metastatic cancer cells are more resistant to anticancer drugs relative to non-metastatic cells (Liang et al., 2002). Conversely, chemoresistant cancer cells are prone to be more invasive and metastatic than sensitive cancer cells. Increasing knowledge on the participation of circRNAs in controlling cancer invasion/metastasis could help to understand the mechanisms underlying circRNA-mediated drug resistance in GI cancer.

EMT is a complicated process in which epithelial cells acquire the characteristics of invasive mesenchymal cells (Du and Shim, 2016). EMT is indispensable for cancer cell invasion and metastasis. The causal linkage between EMT and cancer chemoresistance has been increasingly recognized. Cancer cells undergoing EMT display a chemoresistant phenotype similar to CSCs (Du and Shim, 2016). EMT-driven chemoresistance involves the acquisition of resistance to anticancer agent-induced apoptosis. In addition, the tumor microenvironment (e.g., fibroblasts and hypoxia) is a significant factor contributing to EMT-mediated chemoresistance. Multiple lines of evidence have shown that circRNAs are important regulators of the EMT program, invasion and metastasis of GI cancer cells. Not surprisingly, these circRNAs influence the sensitivity of GI cancer cells to chemotherapy. However, the specific mechanisms by which circRNA-regulated EMT alters drug resistance in GI cancer are largely equivocal, and thus follow-up investigations are required.

### 3.7 Drug Efflux

Adenosine triphosphate (ATP)-binding cassette (ABC) subfamily C member 5 (ABCC5), also known as multidrug resistance protein 5 (MRP5), is a member of the ABC transporter family that regulates the efflux of toxins and drugs (Jansen et al., 2015). The expression of circ_0007031 was positively associated with 5-FU resistance in CRC (He et al., 2020). Circ_0007031 downregulation inhibited CRC cell proliferation and malignancy, and enhanced 5-FU sensitivity. Circ_0007031 functioned as a ceRNA to increase ABCC5 expression by competitively binding to miR-133b in CRC. The effect of circ_0007031 on enhancing drug resistance in CRC was likely attributable to ABCC5-mediated drug efflux. Nevertheless, intracellular 5-FU accumulation in circ_0007031-knockdown CRC cells should be determined in future studies to provide direct evidence supporting the specific role of circ_0007031 in drug efflux.

The induction of drug efflux is a well-characterized mechanism underlying cancer chemoresistance. ABC transporters are a large and common superfamily of proteins that exploit the energy produced by ATP hydrolysis to export various cytotoxic substances from cells (Zappe and Cichna-Markl, 2020). These efflux pumps diminish the intracellular accumulation of anticancer agents and protect cancer cells from chemotherapeutic medications. Reportedly, chemoresistant cancers exhibited alleviated epigenetic inhibition of *MDR1* through promoter hypomethylation and histone acetylation (Sharma et al., 2010; Toth et al., 2012). CircRNAs play a critical role in the epigenetic modulation of gene expression (Zeng et al., 2021). It is intriguing whether circRNAs drive the epigenetic alteration of efflux pump genes. Further efforts are urgently needed to elucidate the epigenetic mechanism by which circRNAs control the expression of drug efflux genes.

### 3.8 Exosomal Circular RNA-Mediated Chemoresistance

Exosomes are nano-sized extracellular biovesicles of endocytic origin that are shed by most types of cells and circulate in body fluids (Chivet et al., 2014). Exosomes carry a variety of molecular and genetic components of their cells of origin, including lipids, proteins and ncRNAs (Chen et al., 2021). These vesicles may transmit multiple signals that affect cancer development and chemoresistance (Wang et al., 2019). Importantly, exosomes transport chemoresistance-relevant ncRNAs between cancer cells. Exosomal circRNAs have been shown to play a key role in mediating drug resistance transfer in GI cancer (Wang H. et al., 2021). The combination of leucovorin (LV) and 5-FU with OXA (FOLFOX) is a first-line therapeutic regimen for CRC (Guan et al., 2020). The microarray profiles of exosomal circRNAs in FOLFOX-resistant and sensitive cells were previously explored (Hon et al., 2019). In total, 139 circRNAs were aberrantly expressed in FOLFOX-resistant CRC cell-derived exosomes, including 105 upregulated and 34 downregulated circRNAs. Among these, circ_0000338 was markedly upregulated in FOLFOX-resistant CRC cell-derived exosomes compared with sensitive cell-originated exosomes. Chemoresistant CRC cell-derived exosomes transported circRNAs into sensitive CRC cells and increased the viability of recipient cells in the presence of 5-FU. Thus, FOLFOX-resistant CRC cell-secreted exosomes conferred drug resistance to sensitive CRC cells via the selective transfer of circRNAs. The loss-of-function study showed that knockdown of circ_0000338 decreased the viability of FOLFOX-resistant CRC cells in the presence of 5-FU. Thus, circ_0000338 might be involved in CRC chemoresistance. The exact roles of circ_0000338 in CRC chemoresistance deserve further study. Moreover, the impact of circ_0000338 on cellular signaling cascades remains to be studied.

The production of ATP through aerobic glycolysis is required for the growth and acquisition of chemoresistance in CRC (Wang X. et al., 2020). The M2 isoform of pyruvate kinase (PKM2) plays an important role in aerobic glycolysis (Chaneton and Gottlieb, 2012). The circRNA ciRS-122 was reported to increase PKM2 expression by decoying miR-122 (Wang X. et al., 2020). Both *in vitro* and *in vivo* studies demonstrated that OXA-resistant CRC cells transferred ciRS-122 to sensitive cells via exosomes, and exosomal ciRS-122 fostered glycolysis and OXA resistance in sensitive cells, as reflected by increases in glucose uptake, lactate, ATP production and tumor growth. In summary, exosomal ciRS-122 weakened drug susceptibility in recipient CRC cells by targeting the miR-122/PKM2 pathway. PKM2 expedites cancer growth, metastasis and chemoresistance by altering cancer cell metabolism or cellular signaling pathways. Blockade of PKM2 activity was shown to repress glycolysis and override drug resistance in cancer (Li et al., 2016). Hence, ciRS-122 might serve as a promising target for sensitizing CRC cells to chemotherapy. The expression of circ-FBXW7 was declined in OXA-resistant CRC tissues and cells compared with sensitive samples (Xu et al., 2021). Exosome-mediated delivery of circ-FBXW7 from normal colon cells to CRC cells reduced drug efflux and conferred chemosensitivity to OXA-resistant CRC cells by reducing the expression of MRP1 and Mcl-1. Exosomal circ-FBXW7 promoted OXA-induced apoptosis by increasing the levels of cleaved caspase-3. It also impeded the migration and invasion of OXA-resistant CRC cells by blocking the EMT program. Overexpression of miR-18b-5p, the downstream target of circ-FBXW7, overturned circ-FBXW7-induced sensitivity to OXA and thus attenuated the anticancer effects of circ-FBXW7. *In vivo* experimental evidence also confirmed that exosomal circ-FBXW7 reversed OXA resistance and inhibited CRC growth partially by tethering miR-18b-5p. Circ-FBXW7 induced chemosensitization of CRC cells by governing drug transport, cell apoptosis and the EMT process. Circ-FBXW7 might have a potential application in CRC therapy. However, additional studies are needed to screen and validate the downstream effectors of the circ-FBXW7/miR-18b-5p regulatory axis.

Circ_0032821, a circRNA that was expressed at high levels in OXA-resistant GC cells, was also associated with GC chemoresistance (Zhong et al., 2021). Circ_0032821 was mainly secreted from GC cells via exosomes. The expression level of exosomal circ_0032821 secreted by resistant cells was significantly higher than that secreted by sensitive cells. OXA-resistant GC cell-derived exosomal circ_0032821 might be incorporated into sensitive GC cells. Exosomal circ_0032821 aggravated OXA resistance, cell proliferation, migration and invasion in sensitive GC cells. Mechanistically, circ_0032821 upregulated the tumor promoter SRY-box transcription factor 9 (SOX9) by sequestering miR-515-5p. Notably, miR-515-5p was previously identified to function as a repressor of GC progression (Wang D. et al., 2020). It was proposed that circ_0032821 modulated cancer progression and the development of OXA resistance in GC through the miR-515-5p/SOX9 axis. Circ-PVT1 was highly expressed in exosomes from serum samples of DDP-resistant GC patients and from DDP-resistant GC cells (Yao W. et al., 2021). YAP1 is a crucial effector of the Hippo signaling pathway and participates in carcinogenesis (Shibata et al., 2018). Circ-PVT1 regulated YAP1 expression by targeting miR-30a-5p. Circ-PVT1 interference reduced LC3-II/I and P-glycoprotein (P-gp) expression but increased P62 expression in DDP-resistant cells. These events led to decreased DDP resistance in DDP-resistant GC cells, which could be reversed by a miR-30a-5p inhibitor or YAP1 overexpression. Thus, circ-PVT1 deletion increased DDP sensitivity in DDP-resistant GC cells by facilitating apoptosis and inhibiting autophagy or invasion through the miR-30a-5p/YAP1 axis. Exosomal circRNAs enhanced chemoresistance of GC cells through promotion of malignancy and cancer progression, inhibition of apoptosis or enhancement of autophagy.

The expression level of circ_0000337 was higher in exosomes from DDP-resistant ESCC cells than in those from sensitive ESCC cells (Zang et al., 2021). Exosomes derived from DDP-resistant ESCC cells induced sensitive cells to develop DDP resistance by delivering circ_0000337. Mechanistic investigation indicated that circ_0000337 interacted with miR-337-3p in ESCC cells, and miR-337-3p overexpression mitigated exosomal circ_0000337-mediated DDP resistance by targeting the oncogenic Janus kinase 2 (JAK2). Coincidentally, xenograft results proved that exosomal circ_0000337 facilitated tumor growth and DDP resistance in ESCC *in vivo*. These results pointed to a potential role of exosomal circ_0000337 in the development of DDP resistance in ESCC.

Collectively, exosomes contribute to spreading chemoresistant phenotypes from chemoresistant cancer cells to chemosensitive cells by delivering circRNAs. Exosomal circRNAs are anticipated to be promising therapeutic targets for the treatment of drug-resistant GI cancer due to their high stability, tissue/cell specificity and functional diversity. Even with these encouraging results, many obstacles must be addressed. Standardized approaches for isolating, collecting and quantifying exosomes and their ncRNA cargos must be developed. Based on accumulating evidence, exosomal circRNAs perform biological functions by decoying miRNAs. It is elusive whether they can act as protein scaffolds or translation templates. The detailed mechanisms associated with the enrichment of circRNAs in exosomes remain largely unknown. Given the low abundance of exosomal circRNAs, much work is required to ascertain the genuine contribution of exosomal circRNAs to the transmission of drug-resistant phenotypes among cancer cells. Finally, clinical studies with large cohorts should be undertaken to clarify the therapeutic potential of exosomal circRNA-based anticancer therapies. Despite their great prospects, there is still a long way to go before turning the promise of exosomal circRNA-based therapeutics into clinical reality.

## 4 Conclusion and Future Perspectives

Chemoresistance has become a major hurdle undermining the efficacy of cancer chemotherapy. It is essential to elucidate the mechanisms associated with cancer chemoresistance, which will accelerate the development of improved therapeutic approaches for cancer. According to the body of evidence described above, circRNAs play key roles in GI cancer chemoresistance. Specifically, circRNAs affect DNA damage repair pathways, cell viability and proliferation, cell death pathways, glucose metabolism, stem cell-like properties, EMT, cell invasion and metastasis, and drug efflux in several GI cancers (Figure 2). The mechanisms underpinning the roles of circRNAs in GI cancer chemoresistance are quite complicated, and concerted research efforts are warranted to obtain a thorough understanding of the relationships between circRNAs and chemoresistance in GI cancer.

The profound impacts of circRNAs on drug resistance in GI cancer make them promising therapeutic targets for GI cancer treatment. However, there are still several issues that need to be addressed before circRNA-based therapeutics can be clinically applied to treat GI cancer. First, comprehensive profiles of the expression patterns of circRNAs between chemoresistant and sensitive GI cancers are needed. Due to the close linkage between aberrantly expressed circRNAs and the drug resistance of GI cancers, screening and identification of key deregulated circRNAs will be conducive to seeking potential therapeutic targets for chemoresistant GI cancer. Moreover, further studies should focus on validating the expression and function of deregulated circRNAs in GI cancer. Second, the mechanisms of action of circRNAs in chemoresistant GI cancer remain largely obscure, and more studies are needed to explain the underlying mechanisms. Remarkably, current studies examining the molecular mechanisms of circRNA-associated chemoresistance have mainly focused on the miRNA sponging function of circRNAs. It is of great importance to substantiate whether circRNAs regulate GI cancer chemoresistance through other mechanisms, including transcriptional modulation or the generation of functional proteins. Extensive investigations should be conducted to completely unveil the mechanisms responsible for the implication of circRNAs in GI cancer chemoresistance. Third, the roles of specific circRNAs in MDR of GI cancer require in-depth research. MDR is defined as the resistance of cancer cells to various chemotherapeutic agents with distinct structures and modes of action (Baguley, 2010). MDR severely weakens the efficacy of clinical chemotherapy in cancer treatment (Bukowski et al., 2020). Nevertheless, relatively few studies have described the role of circRNAs in regulating MDR in GI cancer. It stresses a critical need of the characterization of key circRNAs in GI cancer exhibiting the MDR phenotype. Because circRNAs may target the same molecule or signaling pathway in different cancers, certain circRNAs will likely have an identical regulatory role in various GI cancers exposed to different chemotherapies. Therefore, future studies should be dedicated to discovering common circRNAs in different GI cancers and to defining their contributions to cancer chemotherapy responsiveness, which will expand our comprehension of the explicit mechanisms behind drug resistance in GI cancer.

Finally, the circRNA-associated regulatory axes that are activated during the development of chemoresistance in GI cancer must be systematically delineated. It has been generally accepted that ncRNAs play a momentous role in regulating nearly all cancer hallmarks. Deciphering intricate ncRNA regulatory networks has become a significant critical research direction in the field of oncology. The upstream effectors of circRNAs involved in GI cancer chemoresistance have yet to be identified. The molecules that regulate circRNA biogenesis may be potentially related to the acquisition of drug resistance in GI cancer. Considering the involvement of RBPs in circRNA formation, continuous studies are required to determine whether RBPs can modify drug susceptibility in GI cancer by regulating the abundance of circRNAs. At present, many gaps exist in our knowledge of the mechanisms regulating circRNA biogenesis. Thus, considerable efforts are warranted to delve into the biogenetic process of circRNAs. Significant improvements in the knowledge of circRNA biology will broaden our understanding of the impact of the mechanisms regulating circRNA biogenesis on chemotherapy resistance in cancer. The ceRNA regulatory network plays an important role in cancer pathogenesis. Long noncoding RNAs (LncRNAs) and circRNAs control the expression of diverse mRNAs by acting as ceRNAs for miRNAs. They may compete with the same miRNA response element (MRE) to dominate the expression of miRNA downstream target genes. A large number of reports have indicated a causal relationship between lncRNA dysregulation and chemotherapy responsiveness in cancer. The crosstalk between the circRNA/miRNA/mRNA and lncRNA/miRNA/mRNA pathways is still fairly elusive. The intricate ceRNA regulatory circuits involved in cancer chemoresistance merit intensive investigation. A comprehensive exploration of the circRNA-associated ceRNA network will help to characterize the reciprocal interactions between ncRNAs and mRNAs and provide novel insights into the molecular mechanisms underlying chemoresistance in GI cancer. Even with these challenges, it is also believed that circRNA-based therapeutics could be an effective supplement to conventional treatment to overcome drug resistance in cancer patients.
